# Rhodamine-B for the mark, release, and recapture experiments in gamma-irradiated male *Aedes aegypti* (*Diptera*: *Culicidae*): Persistence, dispersal, and its effect on survival

**DOI:** 10.14202/vetworld.2024.1872-1879

**Published:** 2024-08-24

**Authors:** Hadian Iman Sasmita, Beni Ernawan, Tri Ramadhani, Sunaryo Sunaryo, Mujiyanto Mujiyanto, Alfa Putra Benariva, Yorianta Hidayat Sasaerila

**Affiliations:** 1 Research Center for Radiation Process Technology, National Research and Innovation Agency of Indonesia (BRIN), Jakarta 12440, Indonesia; 2 Research Center for Public Health and Nutrition, National Research and Innovation Agency of Indonesia (BRIN), Cibinong 16915, Indonesia; 3 Department of Biology, Faculty of Science and Technology, Al Azhar Indonesia University, Jakarta 12110, Indonesia

**Keywords:** irradiated males, mark-release-recapture, rhodamine-B, sterile insect technique

## Abstract

**Background and Aim:**

Rhodamine-B (Rh-B) marking shows a great potential for use in mark-release-recapture (MRR) studies for rear-and-release mosquito control strategies, including the radiation-based sterile insect technique. However, its applicability and evaluation in body-stain-irradiated males of *Aedes aegypti* have received little attention. The present study evaluated the use of Rh-B to mark gamma-irradiated male *A. aegypti*.

**Materials and Methods:**

Male *A. aegypti* were irradiated at the pupal stage at a dose of 70 Gy. After emergence, males were fed 0.1, 0.2, 0.3, or 0.4% Rh-B in 10% glucose solution for 4 days. Groups of unirradiated males that received the same feeding treatments were used as control groups. We evaluated the persistence of Rh-B and the longevity of males after Rh-B feeding. Furthermore, the use of Rh-B in irradiated *A. aegypti* for MRR experiments was evaluated at an urban site.

**Results:**

No difference was observed in the Rh-B persistence among all concentrations at the 24-h postmarking period ranging from 91.25 ± 1.61% to 96.25 ± 1.61% and from 90.00 ± 2.28% to 93.13 ± 2.77% for the unirradiated and irradiated groups, respectively. Rh-B persistence significantly decreased over time, and persistence was significantly longer with increased concentrations in both the unirradiated and irradiated groups. Longevity was considerably decreased by Rh-B feeding and irradiation. However, no significant difference in longevity was found among males fed various concentrations of Rh-B. Through MRR experiments, irradiated-Rh-B marked males were mostly detected within a radius of 20 m and 40 m from the center-release point. The mean distance traveled of the released males from the three MRR events was calculated to be 42.6 m.

**Conclusion:**

This study confirms that Rh-B body marking through sugar feeding is applicable for irradiated male *A. aegypti*, with only a slight effect on longevity. Furthermore, considering the significant reduction in persistence over time, further study is needed to assess the impact of this reduction on the calculation of field biological parameters resulting from MRR experiments.

## Introduction

The mark-release-recapture (MRR) experiment to evaluate the biological parameters of radio-sterilized males and population parameters of the targeted species is a prerequisite for sterile insect technique (SIT) field trials against *Aedes* *aegypti* [1]. Survival, dispersal, and mating competitiveness of released sterile males, as well as the target population size, are among the fundamental parameters that need to be thoroughly studied to ensure the success of an SIT program [2]. For instance, several SIT studies on *Aedes* *albopictus* have estimated the survival (average life expectancy [ALE]), dispersal ability (average mean distance traveled [MDT]), mating competitiveness of irradiated and unirradiated males, and the wild population size in preparation for an SIT program [3–5]. In MRR, marked mosquitoes are released into the field and subsequently recaptured at certain times and distances [6].

Guidelines for MRR procedures of *Aedes* mosquitoes are available for SIT study. In the guidelines, fluorescent pigments are used to mark the external organs of males [7]. However, a recent innovation in the marking technique involves the use of rhodamine-B (Rh-B) to mark the body and seminal fluid of *A. aegypti* has been developed to support male-based rear and release strategies [8]. This relatively new marking technique is potentially useful for MRR studies to estimate mosquito movement, mating, and population parameters [9]. The application of Rh-B to mark radio-sterilized males is quite challenging because ionic radiation exposure results in decreased quality of males [10]. Although there were no observed negative side effects of Rh-B marking in wild and *Wolbachia*-infected *A. aegypti* (*w*Mel strain) [8], it is worth evaluating whether the combination of gamma radiation and Rh-B marking may have multiple adverse effects on the released colony. Moreover, alternative techniques for marking in MRR studies need to be explored since several studies have reported the shortcomings of fluorescent powders and their marking procedures [11–15]. After a promising demonstration of the Rh-B marking procedure for MRR studies in *A. aegypti*, the use of Rh-B has been successfully implemented in mating competitiveness and interaction studies in *A. aegypti* [9, 16] and *Anopheles* *gambiae* [17]. However, the applicability of Rh-B marking to gamma-irradiated *A. aegypti* males is yet to be evaluated.

Thus, this study aimed to evaluate the use of Rh-B in gamma-irradiated male *A. aegypti*. Specifically, we assessed the effects of Rh-B concentration on the persistence of the marker in the body of irradiated males and their survivorship. In addition, we investigated the dispersal of irradiated-Rh-B-marked males in the field through MRR experiments. Data resulting from this study provide additional information for developing an alternative marking method for MRR studies as a prerequisite before the field implementation of the SIT program.

## Materials and Methods

### Ethical approval

The experimental protocols, including mosquito colony rearing, gamma irradiation, and MRR experiments, were reviewed by the designated academic board of Al Azhar Indonesia University (No. 002/SK/FST/UAI/VI/2019).

### Study period and location

The study was conducted from December 2018 to March 2019. Mosquito rearing, irradiation, and laboratory experiments were conducted from December 2018 to February 2019 at the insectarium and irradiator unit of BRIN, Jakarta. The MRR experiment was subsequently carried out from February to March 2019 in South Tangerang City, Banten Province.

### Mosquito experimental colony

The *A. aegypti* strain used in this study was a local strain that has been reared at the insectarium of BRIN, Jakarta, since 2017. To obtain males for the experimental colonies, eggs were soaked in aged tap water for hatching. After 24 h, larvae were transferred into a plastic tray (long × wide × tall = 29.5 × 23.0 × 5.0 cm) filled with 1 L aged tap water. The larvae were reared at a density of 3–4 larvae/mL and fed 0.5 g/day Pedigree® dog biscuit (Pedigree®, Mars Petcare Co., Ltd., Bangkok, Thailand) until pupation. Male and female pupae were separated using larval-pupal separator model 5412 (John W. Hock Company, Florida, USA). Male pupae were transferred into plastic cups (10 cm diameter; 8 cm height) containing ± 100 mL aged tap water for treatment.

### Irradiation procedure

Male pupae aged >24 h were placed in a Petri dish (d = 9 cm) containing a small amount of water to create damp conditions. Male pupae were irradiated at a dose of 70 Gy, which induces approximately 98% sterility [18, 19]. The irradiation process was carried out using a Gamma cell 220 irradiator (original version: Atomic Energy of Canada Ltd., Ottawa, Canada; upgraded version: Izotop, Institute of Isotopes Co. Ltd., Budapest, Hungary with a radiation source of Cobalt-60, current activity of 3,549 Curie, and dose rate of 2,581.8 Gy/h). The irradiated pupae were subsequently placed in adult cages measuring 30 × 30 × 30 cm (BugDorm 1®, Mega View Science Co., Ltd., Taichung, Taiwan) for emergence.

### Rh-B solution preparation and marking procedure

The marking procedure of gamma-irradiated male *A. aegypti* was following a previous study by Johnson *et al*. [8] with slight modification. Briefly, Rh-B powder [Sigma-Aldrich, USA, ≥95% dye content (High-performance liquid chromatography)] was dissolved in 10% sugar solution to obtain four concentrations: 0.1, 0.2, 0.3, and 0.4%. Each Rh-B solution was placed in a small plastic cup (4 cm diameter, 1 cm height) equipped with roller cotton for feeding. Rh-B body staining in males was obtained by feeding males *A. aegypti* with the solution for a consecutive 4-day postemergence [8].

### Effects of Rh-B concentration on the persistence and longevity of gamma-irradiated male *A. aegypti*

**Figure-1:**
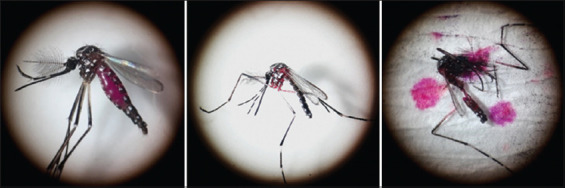
Visibly positive Rh-B bodies in irradiated male *Aedes aegypti* specimen under a stereo microscope (Motic SMZ-171 Series, Kowloon, Hong Kong) at 4× magnification. Left: Rh-B pin the abdomen, thorax, and crushed male body on filter paper. Rh-B=Rhodamine-B.

To evaluate the effects of Rh-B concentration on the persistence of the marker in the irradiated male body and longevity, the following experiments were conducted. For the investigation of various concentrations of Rh-B persistence over time, both unirradiated (control) and gamma-irradiated male *A. aegypti* were placed into an adult cage and provided free access to Rh-B solutions with concentrations of 0.1, 0.2, 0.3, or 0.4% for 4 days. After a consecutive 4-day marking period, the Rh-B solution was replaced with a 10% sugar solution. Persistence was observed in an independent cohort at 24, 48, 72, 96, and 120-h postmarking periods. Forty males were randomly taken from the marking cage and freeze-killed at −20^o^C for 30 min. The presence of Rh-B was then observed individually under a stereomicroscope (Motic SMZ-171 Series, Kowloon, Hong Kong) (Figure-1). The persistence rate was calculated by dividing the number of positive Rh-B males by the total number observed. Each experiment was conducted in four replications.

For longevity measurements, 50 gamma-irradiated or unirradiated (control) male *A. aegypti* were taken from the marking cage for each Rh-B concentration, placed into a 17.5 × 17.5 × 17.5 cm cage (Bugdorm-4M1515, MegaView Science Co., Ltd., Taichung, Taiwan), and continuously supplied with water. Longevity was determined by counting the daily survival (interval 24 h) until all males succumbed to natural mortality [19].

### Release and recapture of gamma-irradiated-Rh-B-marked male *A. aegypti*

**Figure-2:**
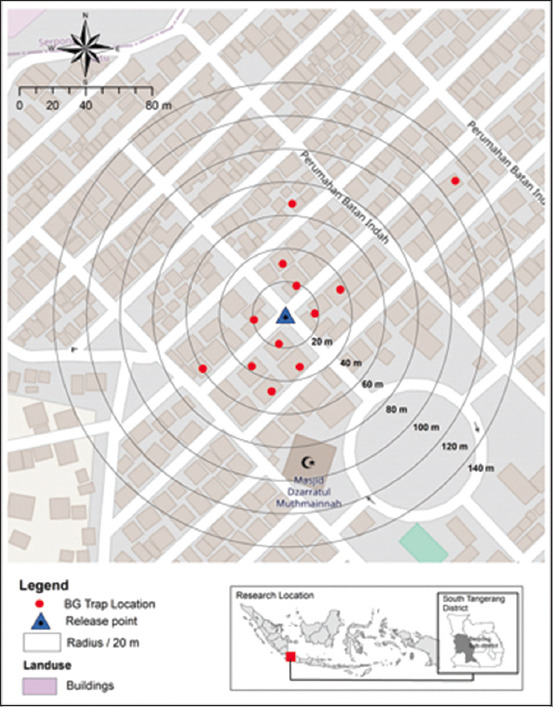
Release points and 12 BG-Sentinel trap placements for release and recapture trials of irradiated Rhodamine-B-marked males *Aedes aegypti*. The virtual annuli were set at a 20-m interval.

Release and recapture experiments were performed in the Batan Indah housing complex (6^o^19’45’’S, 106^o^40’12’’E), South Tangerang City, Banten Province, Indonesia.Twelve adult traps with lures (BG Sentinel-2, Biogents, Regensburg, Germany) were placed indoors at 12 houses (Figure-2). In this experiment, gamma-irradiated male *A. aegypti* were marked using 0.4% Rh-B in 10% sugar solution. The release of the males was conducted 3 times from a single point. Data collection was performed daily, starting 1-day post-release for 5 days by changing the collection bags. Each release event was separated by a 3-day interval. Temperature and humidity at the time when males were released into the study site were recorded using a thermo-hygrometer HT110 (PCE Instruments Hong Kong Ltd., Hong Kong), while wind speed during the day’s recapture was recorded using an anemometer UT-363 (Uni-Trend Technology Co., Ltd. China).

The MDT was calculated by drawing virtual annuli around the release point. The annulus was 20 m apart. A correction factor (CF) was applied to the calculation to accommodate unequal capture densities. Hence, the MDT and CF were determined according to Lillie *et al*. [20] and Morris *et al*. [21], where MDT = Sum (Estimated recapture [ER] × distance) for all annuli/Total number of ER. The ER was defined as ER = (Number of observed recaptures in annulus/Number of traps in annulus) × CF. CF = (Area of annulus/total trapping area) × total number of traps. Distance is defined as inner radius plus outer radius divided by 2.

### Statistical analysis

Data were pooled based on the studied parameters. Before the analysis, persistence data for Rh-B were tested for normality and homogeneity. A general linear model (GLM) full univariate factorial test followed by Tukey’s *post hoc* test was performed to examine the influence of the treatments (i.e., gamma irradiation, Rh-B concentrations, and time postmarking period) on persistence. Longevity was analyzed using Kaplan-Meier survival analysis followed by Mantel-Cox log-rank and Kruskal–Wallis tests. All statistical analysis was performed using Statistical Package for the Social Sciences version 22.0 for Windows (IBM Corp., Armonk, NY, USA).

## Results

### Effects of Rh-B concentration in persistence and longevity of male *A. aegypti*

The GLM analysis showed that Rh-B persistence was significantly affected by Rh-B concentration and time postmarking as a single factor, whereas irradiation had no significant effect. In the two-way interaction, there was no significant effect on the Rh-B persistence between Rh-B, whereas the effect of Rh-B concentration and time postmarking was significantly different depending on the level of time postmarking and irradiation, respectively. Interestingly, in the three-way interaction, the effects of Rh-B concentration, time postmarking, and irradiation factors did not differ from the simple sum of their effects (Table-1). The mean Rh-B persistence at the 24-h postmarking period in all Rh-B concentrations ranged from 91.25 ± 1.61% to 96.25 ± 1.61% and from 90.00 ± 2.28% to 93.12 ± 2.77% for the unirradiated and irradiated male *A. aegypti* groups, respectively, and there was no significant difference among the Rh-B concentrations in each irradiation treatment. In general, Rh-B persistence significantly reduced over time until the 120-h postmarking period, with the value ranging from 3.75 ± 1.61% to 18.12% and from 4.38 ± 2.13% to 25.00 ± 2.28% for the unirradiated and irradiated male groups, respectively (Table-2). In the present study, males fed 0.4% Rh-B resulted in longer persistence of Rh-B both in unirradiated and irradiated groups, whereas lower Rh-B concentrations significantly reduced Rh-B persistence in male *A. aegypti* body markings. However, no significant difference was observed between 0.3 and 0.4% Rh-B concentrations.

**Table-1:** GLM analysis of the effects of gamma irradiation, Rh-B concentration, and time postmarking on Rh-B persistence.

Factor	df	Mean square	F	p-value
Rh-B concentration	3	0.329	30.357	<0.0001
Time postmarking	4	4.560	421.172	<0.0001
Irradiation	1	0.009	0.876	0.351
Rh-B concentration*time postmarking	12	0.020	1.847	0.048
Rh-B concentration*irradiation	3	0.001	0.112	0.953
Time postmarking*irradiation	4	0.037	3.301	0.011
Rh-B concentration*time postmarking*irradiation	12	0.004	0.362	0.974

GLM=General linear model, Rh-B=Rhodamine-B

**Table-2:** Rh-B persistence in unirradiated and irradiated male *A. aegypti* groups at various concentrations and time postmarking periods.

Treatments		Persistence (mean ± SE) (%)				
	
Gamma irradiation	Rh-B concentration	Time postmarking				

		24 h	48 h	72 h	96 h	120 h
Unirradiated	0.1%	91.25 ± 1.61^aA^	65.00 ± 5.10^aB^	39.38 ± 5.14^aC^	11.25 ± 3.89^aD^	3.75 ± 1.61^aD^
	0.2%	92.50 ± 3.23^aA^	65.62 ± 3.44^aB^	43.12 ± 4.25^abC^	21.25 ± 2.17^abD^	7.50 ± 2.28^aD^
	0.3%	94.38 ± 1.20^aA^	72.50 ± 3.68^aB^	57.50 ± 5.20^abB^	28.75 ± 3.31^bC^	13.12 ± 5.98^aC^
	0.4%	96.25 ± 1.61^aA^	75.00 ± 2.28^aB^	62.50 ± 4.21^bB^	35.62 ± 5.44^bC^	18.12 ± 5.72^aC^
Irradiated	0.1%	90.00 ± 2.28^aA^	65.00 ± 5.30^aB^	37.50 ± 5.10^aC^	19.38 ± 3.73^aD^	4.38 ± 2.13^aD^
	0.2%	90.62 ± 3.13^aA^	65.62 ± 3.73^aB^	43.75 ± 4.62^abC^	25.62 ± 5.04^abD^	9.38 ± 2.77^aD^
	0.3%	91.88 ± 2.13^aA^	66.25 ± 5.05^aB^	53.12 ± 3.29^abBC^	42.5 ± 4.89^bC^	23.75 ± 4.39^bD^
	0.4%	93.12 ± 2.77^aA^	70.62 ± 4.38^aB^	58.75 ± 2.17^bBC^	44.38 ± 4.38^bC^	25.00 ± 2.28^bD^

The same lowercase and uppercase letters indicate no significant difference within a column (in each gamma irradiation treatment) and a row, respectively (p < 0.05), Rh-B=Rhodamine-B, *A. aegypti=Aedes aegypti*

**Figure-3:**
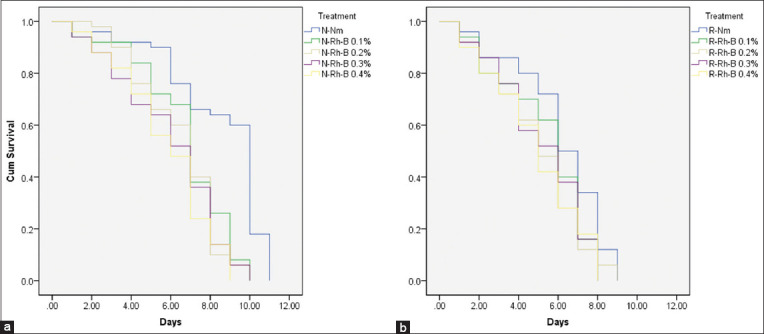
Kaplan-Meier survival curves of (a) unirradiated and (b) gamma-irradiated male *Aedes aegypti* postmarking periods at various Rh-B concentrations. N-Nm, N-Rh-B, R-Nm, and R-Rh-B represent unirradiated-unmarked, unirradiated-marked with Rh-B (0.1%–0.4%), irradiated-unmarked, and irradiated-marked with Rh-B (0.1%–0.4%), respectively. Rh-B=Rhodamine-B.

The longevity of the unirradiated and irradiated male *A. aegypti* postmarking periods is presented in Figure-3 and Table-3. There was a significant difference in the longevity of unirradiated *A. aegypti* males between the unmarked and marked groups regardless of Rh-B concentrations. The mean longevity of unirradiated-unmarked males was 8.50 ± 0.375 days, whereas the unirradiated-marked groups were reduced with increasing Rh-B concentration ranging from 6.74 ± 0.332 to 5.80 ± 0.327 days (Table-3). However, this reduction was not significantly different (long-rank test, p < 0.05). Similar results were found in the longevity of irradiated male *A. aegypti*. There was a significant difference between the unmarked and marked groups, except for the 0.1% Rh-B group. The mean longevity of the irradiated-unmarked males was 6.16 ± 0.32 days, whereas the irradiated-marked groups tended to decrease with increasing Rh-B concentration ranging from 5.38 ± 0.317 to 4.9 ± 0.322 days (Table-3). However, this reduction was not significantly different (log-rank test, p < 0.05). In addition, the irradiation factor significantly affected the longevity of male *A. aegypti* (long-rank test, p < 0.05).

**Table-3:** Longevity of the postmarking period of male *A. aegypti* at various Rh-B concentrations.

Treatments		Longevity (mean ± SE) (95% CI) (days)

Gamma irradiation	Rh-B concentration	
Unirradiated	Unmarked	8.5 ± 0.375 (7.765–9.235) ^aA^
	0.1%	6.74 ± 0.332 (6.09–7.39) ^bA^
	0.2%	6.46 ± 0.293 (5.886–7.034) ^bA^
	0.3%	6.0 ± 0.365 (5.285–6.715) ^bA^
	0.4%	5.80 ± 0.327 (5.159–6.441) ^bA^
Irradiated	Unmarked	6.16 ± 0.32 (5.533–6.787) ^aB^
	0.1%	5.38 ± 0.317 (4.759–6.001) ^abB^
	0.2%	5.0 ± 0.323 (4.366–5.634) ^bB^
	0.3%	5.18 ± 0.314 (4.565–5.795) ^bB^
	0.4%	4.9 ± 0.322 (4.268–5.532) ^bB^

CI=Confidence interval. Pairwise comparisons of longevity were performed using log-rank (Mantel-Cox) and Kruskal–Wallis tests. The same lowercase and uppercase superscript letters indicate no significant difference in a column within irradiation treatment and rows within the same Rh-B concentration, respectively (p < 0.05), *A. aegypti=Aedes aegypti*, Rh-B=Rhodamine-B, SE=Standard Error

### Release and recapture of irradiated Rh-B-labeled males

The temperature and humidity were 33.08°C and 77.58%, 31.38°C and 72.14%, and 31.9°C and 78.71% during the 1^st^, 2^nd^, and 3^rd^ day of release, respectively. The wind speeds were between 0.4 and 2.2 m/s. The wind speed was averaged at 1.028, 1.024, and 1.013 m/s in the 1^st^, 2^nd^, and 3^rd^ releases, respectively.

**Figure-4:**
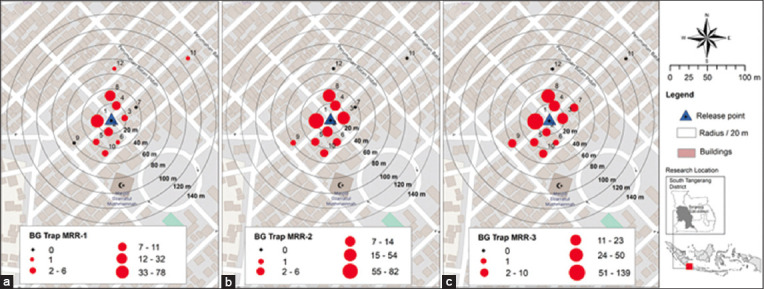
Number of released males recaptured in 5 consecutive days after release in (a) 1^st^, (b) 2^nd^, and (c) 3^rd^ release events.

A total of 14,570 irradiated Rh-B-marked males were released, with 4,789, 4,884, and 4,897 males released during the 1^st^, 2^nd^, and 3^rd^ events, respectively. The percentage of recaptured males over a 5-day period was between 3.12, 4.54, and 5.15% (Table-4). During the first release event, 105 out of 149 released males were recaptured in traps within a radius of 20 m from the release point. In this release, a male was detected in the furthest trap installed between a radius of 120 and 140 m. In the second event, released males were recaptured within a 20–60 m radius, with 162 released males found in traps within a 20 m radius. Similar to the second event, all released males from the third event were captured within a radius of 20–60 m (Figure-4).

**Table-4:** Release and recapture of Rh-B-irradiated male *A. aegypti*.

Release events	No. of males released	No. of males recaptured	The percentage of males recaptured
1^st^	4789	149	3.12%
2^nd^	4884	222	4.54%
3^rd^	4897	252	5.15%

*A. aegypti=Aedes aegypti*, Rh-B=Rhodamine-B

The movement of irradiated Rh-B-marked *A. aegypti* males was estimated to be up to 42.6 m on average. Specifically, for the first, second, and third release events, MDTs were calculated up to 62.3, 24.3, and 27.4 m, respectively.

## Discussion

In this study, we aimed to evaluate the Rh-B marking procedure in irradiated male *A. aegypti*, which may be useful for the future development of the rear-and-release mosquito control strategies, including the classical SIT. We found that the Rh-B marking procedure by Johnson *et al*. [8] was reproducible in irradiated male *A. aegypti*. However, the general persistence of body staining in irradiated males was lower than that observed in wild-type and *w*Mel *A. aegypti* males [8]. Increased metabolism has been reported in gamma-sterilized males [22] and may reduce the persistence of Rh-B staining [17]. Although inspecting the marker under a fluorescent microscope greatly improved the percentage of males positive for Rh-B [8], the gradual loss of Rh-B marking over time is a shortcoming of this technique. Losing the mark during the spell of recapture may affect the estimation of entomological parameters, such as the total population, probability of daily survival, and flight range. Taking the Petersen method as an example, the number of marked individuals captured is one of the components in estimating the total population, in which any inaccurate inputs may lead to an over- or underestimation value [23]. This may be a potential limitation of Rh-B marking because of the ALE of sterile male *A. aegypti* was reported as 3.76 days [24]. A long-lasting marking technique that can last over life expectancy is favorable to ensure that the efficacy of the marking method remains high.

Persistence is an essential attribute of MRR studies; hence, it is challenging to develop a marking method that maintains long-lasting persistence without any negative impact on sterile males. In this study, the persistence of Rh-B staining in the male *A. aegypti* body at 24 h postmarking period with all Rh-B concentrations ranged from 91.25% to 96.25% and 90.00% to 93.13% for the unirradiated and irradiated groups, respectively. No significant difference was observed among the Rh-B concentration groups. We found that Rh-B persistence at all concentrations was significantly reduced over time; however, the highest Rh-B concentration (0.4%) exhibited the longest persistence. A previous study by Trewin *et al*. [9] reported that a Rh-B concentration of 0.4% was used in an MRR study of *A. aegypti* in North Queensland, Australia. Similar results were reported in a previous study by Johnson *et al*. [8] in which higher Rh-B concentrations (0.4% and 0.8%) resulted in long-lasting persistence in body tissue and seminal fluid of male *A. aegypti* for 72-h postmarking observation; however, our persistence reduction was lower. Several factors potentially affect this difference, including the metabolic rate, strain, and environmental conditions. Considering persistence reduction, a comprehensive study is needed to develop a standard Rh-B marking method for mosquito MRR studies. In addition, it is necessary to assess the association between persistence reduction and the effectiveness of MRR.

Longevity, which is correlated to survivability of gamma-irradiated and Rh-B-marked male *A. aegypti*, is one of the main quality parameters for the success of SIT programs [25]. The results of the present study demonstrate that Rh-B in 10% sugar solution at all concentrations was detrimental to unirradiated and irradiated male survivorship. However, there was no difference among the Rh-B concentrations. Similar observations have been observed for both male and female *A. gambiae* [17] but were not observed in wild type and *w*Mel *A. aegypti* [8], tobacco budworm moths *Heliothis* *virescens* [26], sand flies [27], and *Culex* mosquitoes [28, 29]. Various factors may play important roles in the significant decrease in irradiated Rh-B-marked male survival, including the concentration of Rh-B, mosquito strain, and irradiation treatment. Although Rh-B 0.4% worked well in wild and *w*Mel *A. aegypti* culture, strain-specific tolerance to the dye may exist. A lower concentration of Rh-B (0.2%) was used to investigate the mating competitiveness of the *Wolbachia*-infected line *A. aegypti* [16], whereas different mortality rates were reported between *A. coluzzii* (Banfora strain) and *A. gambiae* (Kisumu strain) after 72 h of 0.2% Rh-B exposure [17]. Hence, optimization of Rh-B concentrations for specific mosquito strains is strongly suggested before application.

There was an obvious interaction between Rh-B feeding and gamma irradiation, which slightly reduced longevity compared with individual assessments. This result was expected because Rh-B or gamma irradiation alone reduces longevity. Reduced longevity, regardless of ray exposure, in irradiated male mosquitoes was reported in previous studies by Yamada *et al*. [30], Bond *et al*. [31], Du *et al*. [32], Balestrino *et al*. [33], and Ernawan *et al*. [34]. Somatic damage that often occurs during ionic irradiation may reduce longevity in mosquitoes [35]. The irradiation process induces the bonding of atoms in the molecules in the cell, resulting in the formation of a single radical ion. Free radicals damage more cells, including somatic cells, and the work system can cause DNA mutations [36, 37]. Rh-B has a photodynamic ability, in which the molecules can interact with light and catalyze the reaction of changing triplet oxygen (^3^O_2_) to reactive oxygen such as cytotoxic singlet oxygen (^1^O_2_). Singlet oxygen can oxidize molecules and compounds in cells, causing cell damage [38, 39].

Despite the reduction in persistence and longevity compared with unirradiated males, the recapture rates of irradiated Rh-B-marked males were comparable with those of other MRR studies on other species, using different marking agents, and in various ecotypes. In this study, the recapture rates 5-day post-release were between 3.12% and 5.15%. This is similar to the recapture rates observed for irradiated male *A. albopictus* marked with fluorescent dust [3, 5] and for Rh-B marked wild-type male *A. aegypti* [9]. Our released males were mostly dispersed within a radius of 40 m (Figure-2). This was in accordance with the average MDT of irradiated Rh-B-marked males, which was calculated at 42.6 m. This average MDT was shorter than the MDT of wild Rh-B-marked *A. aegypti* and dusted radio-sterilized *A. aegypti*, which were estimated to be between 126.7 and 310.5 m [9] and 77.3 m [24], respectively. However, the shorter average MDT in our study relative to the two other studies is not a clear indication of the reduced flight ability of irradiated Rh-B-marked male *A. aegypti*, since the MDT was associated with various variables, such as trap placement and time of mosquito release [40].

## Conclusion

The results revealed that higher Rh-B concentrations (0.4%) resulted in long-lasting persistence in male *A. aegypti*, which ranged from 96.25%–18.13% to 93.13%–25.00% in the unirradiated and irradiated groups, respectively. We found that Rh-B was detrimental to the longevity of unirradiated and irradiated male *A. Aegypti;* however, no significant difference was found among the concentrations. According to the data presented here, we found 0.4% Rh-B to be an appropriate concentration for the MRR field experiment on gamma-sterilized male *A. aegypti*, resulting in an average MDT of 42.6 m. However, considering the significant reduction in Rh-B persistence over time, further studies are needed to assess this correlation with the effectiveness of the MRR study in the SIT program.

## Authors’ Contributions

HIS, BE, and YHS: Conceptualization, methodology, and validation. TR, SS, MM, and APB: Investigation and data curation. HIS and BE: Data interpretation and drafted and revised the manuscript. All authors have read, reviewed, and approved the final manuscript.
